# Crystal structure of 4-formyl-2-nitro­phenyl 4-chloro-2-nitro­benzoate

**DOI:** 10.1107/S205698901502006X

**Published:** 2015-11-14

**Authors:** Rodolfo Moreno-Fuquen, Geraldine Hernández, Javier Ellena, Carlos A. De Simone, Juan C. Tenorio

**Affiliations:** a Departamento de Química, Facultad de Ciencias Naturales y Exactas, Universidad del Valle, Apartado 25360, Santiago de Cali, Colombia; b Instituto de Física de São Carlos, IFSC, Universidade de São Paulo, USP, São Carlos, SP, Brazil

**Keywords:** crystal structure, ester, hydrogen bonding, 4-chloro-2-nitro­benzoate

## Abstract

In the title compound, C_14_H_7_ClN_2_O_7_, the central ester moiety is essentially planar, with an r.m.s. deviation of 0.0113 Å. The ester group is twisted away from the chloro- and formyl-substituted rings by 84.60 (9) and 88.55 (9)°, respectively. The crystal packing shows inter­molecular C—H⋯O inter­actions. These inter­actions generate *R*_2_^2^(20) and *R*_4_^4^(22) edge-fused rings parallel to (20-2).

## Related literature   

For related structures, see: Moreno-Fuquen *et al.* (2013 ▸, 2014 ▸). For hydrogen-bond details, see: Nardelli (1995 ▸).
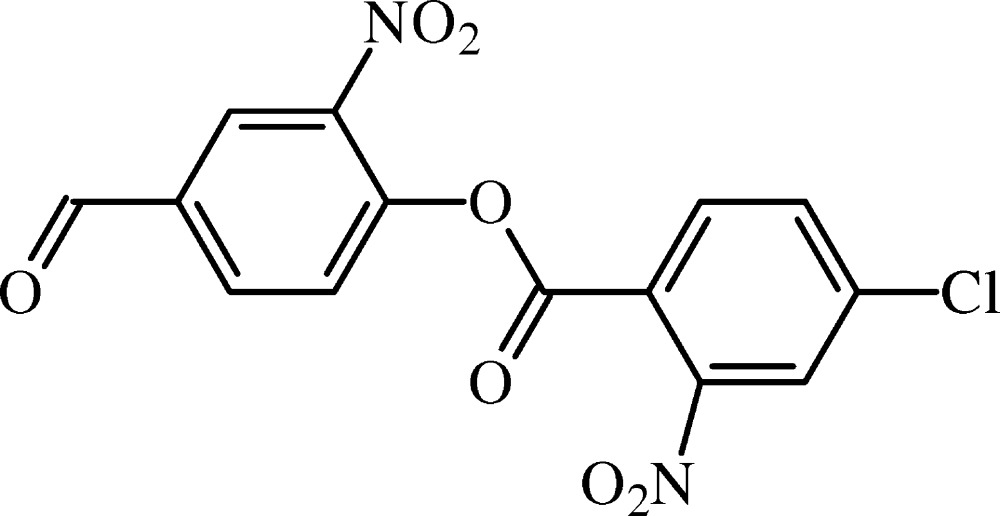


## Experimental   

### Crystal data  

C_14_H_7_ClN_2_O_7_
*M* *_r_* = 350.67
Triclinic 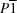

*a* = 7.7366 (2) Å
*b* = 7.9480 (2) Å
*c* = 12.6539 (5) Å
α = 90.0655 (11)°
β = 100.3204 (11)°
γ = 104.0633 (12)°
*V* = 741.75 (4) Å^3^
*Z* = 2
Mo *K*α radiation
μ = 0.30 mm^−1^
*T* = 295 K
0.76 × 0.13 × 0.06 mm

### Data collection  

Nonius KappaCCD diffractometer
5322 measured reflections
3018 independent reflections
2177 reflections with *I* > 2σ(*I*)
*R*_int_ = 0.046

### Refinement  

*R*[*F* ^2^ > 2σ(*F* ^2^)] = 0.060
*wR*(*F* ^2^) = 0.194
*S* = 1.03
3018 reflections
221 parameters
H atoms treated by a mixture of independent and constrained refinement
Δρ_max_ = 0.59 e Å^−3^
Δρ_min_ = −0.37 e Å^−3^

### 

Data collection: *COLLECT* (Nonius, 2000 ▸); cell refinement: *SCALEPACK* (Otwinowski & Minor, 1997 ▸); data reduction: *DENZO* (Otwinowski & Minor, 1997 ▸) and *SCALEPACK*; program(s) used to solve structure: *SIR2014* (Burla *et al.*, 2015 ▸); program(s) used to refine structure: *SHELXL2014* (Sheldrick, 2015 ▸); molecular graphics: *ORTEP-3 for Windows* (Farrugia, 2012 ▸) and *Mercury* (Macrae *et al.*, 2006 ▸); software used to prepare material for publication: *WinGX* (Farrugia, 2012 ▸).

## Supplementary Material

Crystal structure: contains datablock(s) I. DOI: 10.1107/S205698901502006X/hg5461sup1.cif

Structure factors: contains datablock(s) I. DOI: 10.1107/S205698901502006X/hg5461Isup2.hkl

Supporting information file. DOI: 10.1107/S205698901502006X/hg5461Isup3.cml

**Fig. 1.**
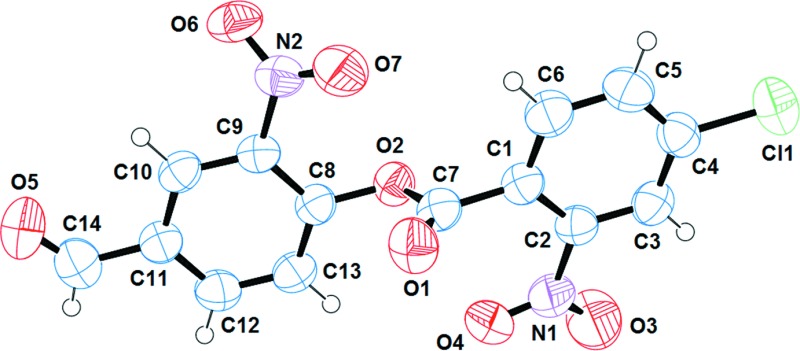
The molecular structure of (I) with displacement ellipsoids drawn at the 50% probability level. H atoms are shown as spheres of arbitrary radius.

. DOI: 10.1107/S205698901502006X/hg5461fig1.tif
The mol­ecular structure of (I) with displacement ellipsoids drawn at the 50% probability level. H atoms are shown as spheres of arbitrary radius.

**Fig. 2.**
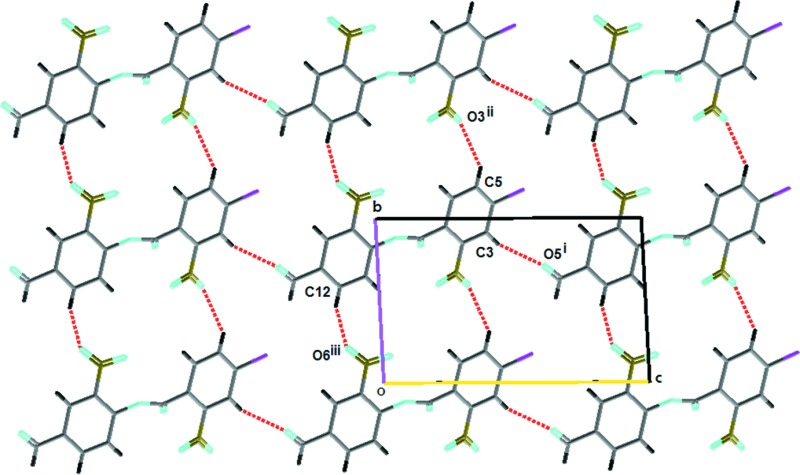
Part of the crystal structure of (I), showing the formation of R22(20) and R44(22) edge-fused rings parallel to (202). [Symmetry codes: (i) x+1,+y,+z+1; (ii) x,+y+1,+z; (iii) x,+y-1,+z ].

. DOI: 10.1107/S205698901502006X/hg5461fig2.tif
Part of the crystal structure of (I), showing the formation of 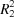
(20) and 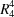
(22) edge-fused rings parallel to (20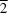). [Symmetry codes: (i) x+1,+y,+z+1; (ii) x,+y+1,+z; (iii) x,+y-1,+z] .

**Fig. 3.**
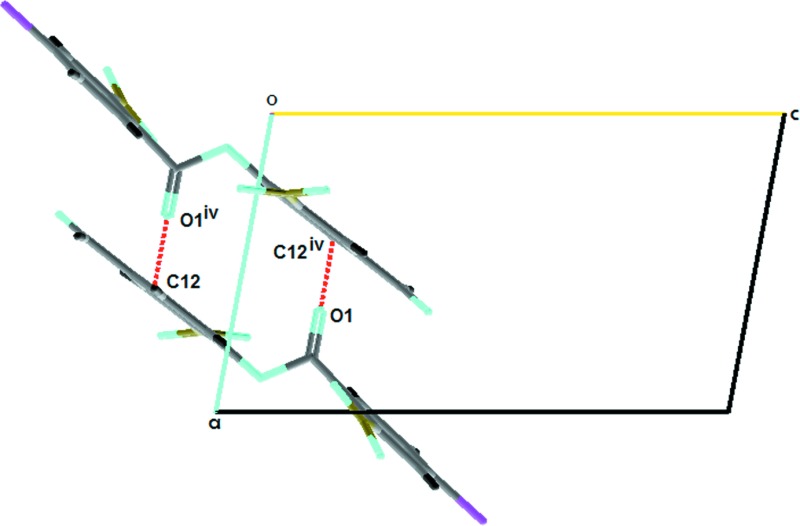
Part of the crystal structure of (I), showing the formation of dimers along [100]. [Symmetry codes: (iv) -x+1,-y+1,-z].

. DOI: 10.1107/S205698901502006X/hg5461fig3.tif
Part of the crystal structure of (I), showing the formation of dimers along [100]. [Symmetry codes: (iv) −x+1,-y+1,-z].

CCDC reference: 1432812

Additional supporting information:  crystallographic information; 3D view; checkCIF report

## Figures and Tables

**Table 1:** Hydrogen-bond geometry (Å, °)

*D*—H⋯*A*	*D*—H	H⋯*A*	*D*⋯*A*	*D*—H⋯*A*
C3—H3⋯O5^i^	0.93	2.44	3.289 (3)	151
C5—H5⋯O3^ii^	0.93	2.42	3.212 (4)	143
C12—H12⋯O6^iii^	0.93	2.58	3.317 (3)	137
C12—H12⋯O1^iv^	0.93	2.68	3.476 (3)	145

Symmetry codes: (i) 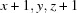; (ii) 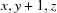; (iii) 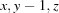; (iv) 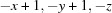.

## supplementary crystallographic information

### S1. Comment

The title compound 4-Formyl-2-nitrophenyl 4-chloro-2-nitrobenzoate (FClNB) (I), is part of a series of studies on the structural properties of the formyl nitro aryl benzoates developed by our research group. Of the many formyl aryl derivative systems studied in our group, can be highlighted the 4-formyl-2-nitrophenyl 4-bromo benzoate (FBrB) (Moreno-Fuquen *et al.*, 2013) and 4-Formyl-2-nitrophenyl benzoate (FB) (Moreno-Fuquen *et al.*, 2014) such as those closest to (I). The molecular structure of (I) is shown in Fig. 1. Bond lengths and bond angles of central ester segment show marked similarity with (FBrB) and (FB). The central ester moiety, C1-C7(O1)-O2-C8, is essentially planar with a r.m.s deviation of fitted atoms of 0.0113 Å. The ester group is twisted away from the chloro and formyl rings by 84.60 (9)° and 88.55 (9)°, respectively.The nitro groups form dihedral angles with the chloro and formyl rings to which it is attached of 11.6 (2)% and 35.03 (8)°. The nitro groups of different rings are *anti* to each other. Comparing (I) with the two aforementioned similar structures, reveals that significant differences in bond lengths and bond angles are not observed. The crystal packing shows no classical hydrogen bonds and it is stabilized by weak C-H···O intermolecular interactions. The C3-H3···O5^i^, C5-H5···O3^ii^ and C12-H12···O6^iii^ hydrogen bond interactions are responsible for crystal growth parallel to (2 0 \-2) (see Fig 2). The C3 atom at (x,y,z) acts as hydrogen bond donor to formyl O5 atom at (i= x+1,+y,+z+1), the C5 atom acts as hydrogen bond donor to O3 atom of the nitro group at (ii= x,+y+1,+z) and the C12 atom acts as hydrogen bond donor to O6 atom of the nitro group at (iii= x,+y-1,+z). These interactions generate *R*_2_^2^(20) and *R*_4_^4^(22) edge-fused rings (see Table 1, Nardelli, 1995). Complement crystal growth, C12-H12···O1 interactions, wherein molecules running parallel to (2 0 2), intertwine with other molecules, forming dimers along [100] (see Fig. 3).

### S2. Experimental

The title molecule was obtained through a two-step reaction: 4-Chloro-2-nitrobenzoic acid (0.200 g, 0.992 mmol) was refluxed with thionyl chloride (5 mL) in acetonitrile for an hour. Then, the thionyl chloride was distilled to purify the 4-chloro-2-nitro benzoyl chloride obtained as a pale-yellow translucent liquid. The same reaction flask was rearranged and an equimolar solution of 4-hydroxy-3-nitrobenzaldehyde (0.166 g, 0.992 mmol) in acetonitrile was dropped inside it with 0.03 mL of pyridine. The reaction mixture was taken to room temperature with constant stirring for about an hour. A shiny yellow solid was obtained after leaving the solvent to evaporate. Yellow crystals; m.p 417 (1) K.

### S3. Refinement

All H-atoms were located from difference maps and were positioned geometrically [C—H = 0.93 Å for aromatic and were refined using a riding-model approximation with *U*_iso_(H) constrained to 1.2 times *U*_eq_ of the respective parent atom. Formyl H141 atom was found from fourier difference maps and its coordinates refined freely.

### Crystal data

**Table:**

C_14_H_7_ClN_2_O_7_	*F*(000) = 356
*M**_r_* = 350.67	*D*_x_ = 1.570 Mg m^−^^3^
Triclinic, *P*1	Melting point: 417(1) K
*a* = 7.7366 (2) Å	Mo *K*α radiation, λ = 0.71073 Å
*b* = 7.9480 (2) Å	Cell parameters from 4404 reflections
*c* = 12.6539 (5) Å	θ = 2.9–26.4°
α = 90.0655 (11)°	µ = 0.30 mm^−^^1^
β = 100.3204 (11)°	*T* = 295 K
γ = 104.0633 (12)°	Needle, colourless
*V* = 741.75 (4) Å^3^	0.76 × 0.13 × 0.06 mm
*Z* = 2	

### Data collection

**Table:**

Nonius KappaCCD diffractometer	2177 reflections with *I* > 2σ(*I*)
Radiation source: fine-focus sealed tube	*R*_int_ = 0.046
Graphite monochromator	θ_max_ = 26.4°, θ_min_ = 2.9°
CCD rotation images, thick slices scans	*h* = −9→9
5322 measured reflections	*k* = −9→8
3018 independent reflections	*l* = −15→15

### Refinement

**Table:**

Refinement on *F*^2^	Primary atom site location: structure-invariant direct methods
Least-squares matrix: full	Secondary atom site location: difference Fourier map
*R*[*F*^2^ > 2σ(*F*^2^)] = 0.060	Hydrogen site location: inferred from neighbouring sites
*wR*(*F*^2^) = 0.194	H atoms treated by a mixture of independent and constrained refinement
*S* = 1.03	*w* = 1/[σ^2^(*F*_o_^2^) + (0.1103*P*)^2^ + 0.1814*P*] where *P* = (*F*_o_^2^ + 2*F*_c_^2^)/3
3018 reflections	(Δ/σ)_max_ < 0.001
221 parameters	Δρ_max_ = 0.59 e Å^−^^3^
0 restraints	Δρ_min_ = −0.37 e Å^−^^3^

### Special details

**Table:**

Experimental. IR spectra was recorded on a FT—IR SHIMADZU IR-Affinity-1 spectrophotometer. IR (KBr), cm^-1^, 3449 and 3090 (aromatic C-H); 1767 (ester, C=O); 1216 (ester C-O); 1040 (ester C8-O6); 1706 (benzaldehyde C=O); 1539, 1349 (nitro-NO_2_ aryl ring); 1487, 1277 (nitro-NO_2_ acyl ring); 1090 (C=C): 754 (C-Cl).
Geometry. All esds (except the esd in the dihedral angle between two l.s. planes) are estimated using the full covariance matrix. The cell esds are taken into account individually in the estimation of esds in distances, angles and torsion angles; correlations between esds in cell parameters are only used when they are defined by crystal symmetry. An approximate (isotropic) treatment of cell esds is used for estimating esds involving l.s. planes.
Refinement. Refinement of *F*^2^ against ALL reflections. The weighted *R*-factor *wR* and goodness of fit *S* are based on *F*^2^, conventional *R*-factors *R* are based on *F*, with *F* set to zero for negative *F*^2^. The threshold expression of *F*^2^ > σ(*F*^2^) is used only for calculating *R*-factors(gt) *etc*. and is not relevant to the choice of reflections for refinement. *R*-factors based on *F*^2^ are statistically about twice as large as those based on *F*, and *R*- factors based on ALL data will be even larger.

### Fractional atomic coordinates and isotropic or equivalent isotropic displacement parameters (Å^2^)

**Table:**

	*x*	*y*	*z*	*U*_iso_*/*U*_eq_	
Cl1	1.36368 (11)	1.17195 (11)	0.56085 (6)	0.0863 (3)	
O2	0.8864 (2)	0.8852 (2)	0.07551 (13)	0.0561 (4)	
O6	0.7439 (2)	1.2200 (2)	−0.13342 (17)	0.0714 (5)	
O1	0.6563 (2)	0.8349 (3)	0.16669 (15)	0.0732 (6)	
N2	0.7314 (3)	1.1215 (3)	−0.06000 (19)	0.0578 (5)	
C3	1.1840 (4)	0.9146 (3)	0.4150 (2)	0.0606 (6)	
H3	1.2487	0.8444	0.4551	0.073*	
C10	0.5786 (3)	0.8747 (3)	−0.18505 (19)	0.0531 (5)	
H10	0.5311	0.9505	−0.2307	0.064*	
O4	0.9084 (3)	0.5985 (2)	0.22127 (17)	0.0810 (6)	
C9	0.6900 (3)	0.9348 (3)	−0.08815 (18)	0.0482 (5)	
C2	1.0593 (3)	0.8503 (3)	0.3237 (2)	0.0555 (6)	
C1	0.9590 (3)	0.9493 (3)	0.26146 (18)	0.0526 (6)	
C13	0.7251 (4)	0.6489 (3)	−0.0490 (2)	0.0625 (6)	
H13	0.7755	0.5736	−0.0044	0.075*	
C11	0.5383 (3)	0.6984 (3)	−0.21338 (19)	0.0566 (6)	
C8	0.7624 (3)	0.8225 (3)	−0.01961 (19)	0.0512 (5)	
N1	1.0326 (4)	0.6653 (3)	0.2922 (2)	0.0769 (7)	
C4	1.2098 (3)	1.0881 (3)	0.4452 (2)	0.0593 (6)	
C12	0.6122 (4)	0.5878 (3)	−0.1456 (2)	0.0649 (7)	
H12	0.5853	0.4703	−0.1654	0.078*	
C6	0.9911 (4)	1.1222 (3)	0.2933 (2)	0.0657 (7)	
H6	0.9285	1.1935	0.2527	0.079*	
C5	1.1161 (4)	1.1901 (3)	0.3853 (2)	0.0686 (7)	
H5	1.1359	1.3065	0.4062	0.082*	
O7	0.7490 (3)	1.1674 (3)	0.03363 (18)	0.0876 (7)	
C7	0.8143 (3)	0.8800 (3)	0.1663 (2)	0.0557 (6)	
O5	0.3385 (4)	0.7111 (4)	−0.37667 (18)	0.1035 (8)	
C14	0.4168 (4)	0.6297 (5)	−0.3168 (3)	0.0772 (8)	
O3	1.1442 (5)	0.5914 (3)	0.3367 (3)	0.1369 (13)	
H141	0.410 (5)	0.511 (5)	−0.334 (3)	0.108 (12)*	

### Atomic displacement parameters (Å^2^)

**Table:**

	*U*^11^	*U*^22^	*U*^33^	*U*^12^	*U*^13^	*U*^23^
Cl1	0.0856 (6)	0.0964 (6)	0.0681 (5)	0.0142 (4)	0.0024 (4)	−0.0178 (4)
O2	0.0450 (8)	0.0656 (10)	0.0572 (10)	0.0139 (7)	0.0080 (7)	0.0066 (7)
O6	0.0663 (11)	0.0533 (10)	0.0999 (14)	0.0223 (8)	0.0190 (10)	0.0204 (10)
O1	0.0483 (10)	0.0982 (14)	0.0721 (12)	0.0133 (9)	0.0148 (8)	0.0026 (10)
N2	0.0483 (10)	0.0498 (11)	0.0785 (14)	0.0166 (8)	0.0136 (9)	0.0053 (10)
C3	0.0641 (14)	0.0626 (15)	0.0569 (14)	0.0214 (12)	0.0082 (11)	0.0086 (11)
C10	0.0494 (12)	0.0564 (13)	0.0582 (13)	0.0197 (10)	0.0132 (10)	0.0118 (10)
O4	0.0919 (14)	0.0577 (11)	0.0816 (14)	0.0053 (10)	0.0037 (11)	−0.0034 (10)
C9	0.0442 (11)	0.0452 (11)	0.0592 (13)	0.0149 (9)	0.0149 (10)	0.0058 (9)
C2	0.0608 (13)	0.0485 (12)	0.0578 (13)	0.0155 (10)	0.0104 (11)	0.0047 (10)
C1	0.0491 (12)	0.0541 (13)	0.0566 (13)	0.0159 (10)	0.0106 (10)	0.0060 (10)
C13	0.0693 (15)	0.0505 (13)	0.0694 (16)	0.0229 (11)	0.0062 (12)	0.0101 (11)
C11	0.0574 (13)	0.0560 (14)	0.0584 (14)	0.0161 (11)	0.0124 (11)	0.0032 (10)
C8	0.0449 (11)	0.0550 (13)	0.0552 (13)	0.0151 (10)	0.0091 (9)	0.0070 (10)
N1	0.0989 (18)	0.0537 (13)	0.0752 (16)	0.0240 (13)	0.0014 (14)	0.0101 (11)
C4	0.0607 (14)	0.0613 (14)	0.0554 (13)	0.0121 (11)	0.0139 (11)	−0.0029 (11)
C12	0.0722 (16)	0.0493 (13)	0.0728 (16)	0.0169 (12)	0.0101 (13)	0.0017 (12)
C6	0.0697 (15)	0.0599 (15)	0.0719 (17)	0.0282 (12)	0.0078 (13)	0.0054 (12)
C5	0.0775 (17)	0.0524 (14)	0.0781 (17)	0.0197 (12)	0.0149 (14)	−0.0040 (12)
O7	0.1177 (18)	0.0639 (12)	0.0821 (14)	0.0263 (11)	0.0157 (13)	−0.0101 (10)
C7	0.0505 (13)	0.0568 (13)	0.0626 (14)	0.0177 (10)	0.0112 (11)	0.0081 (10)
O5	0.1107 (18)	0.121 (2)	0.0679 (14)	0.0290 (16)	−0.0113 (13)	0.0103 (13)
C14	0.0740 (18)	0.080 (2)	0.0723 (19)	0.0134 (16)	0.0074 (15)	−0.0048 (16)
O3	0.186 (3)	0.0732 (15)	0.137 (2)	0.0626 (18)	−0.048 (2)	−0.0010 (15)

### Geometric parameters (Å, º)

**Table:**

Cl1—C4	1.730 (3)	C2—N1	1.478 (3)
O2—C7	1.360 (3)	C1—C6	1.382 (3)
O2—C8	1.404 (3)	C1—C7	1.491 (3)
O6—N2	1.217 (3)	C13—C8	1.376 (3)
O1—C7	1.189 (3)	C13—C12	1.379 (4)
N2—O7	1.213 (3)	C13—H13	0.9300
N2—C9	1.469 (3)	C11—C12	1.380 (3)
C3—C2	1.373 (3)	C11—C14	1.484 (4)
C3—C4	1.388 (4)	N1—O3	1.216 (3)
C3—H3	0.9300	C4—C5	1.359 (4)
C10—C9	1.377 (3)	C12—H12	0.9300
C10—C11	1.391 (3)	C6—C5	1.387 (4)
C10—H10	0.9300	C6—H6	0.9300
O4—N1	1.205 (3)	C5—H5	0.9300
C9—C8	1.390 (3)	O5—C14	1.177 (4)
C2—C1	1.386 (3)	C14—H141	0.95 (4)
			
C7—O2—C8	115.60 (17)	C13—C8—C9	120.0 (2)
O7—N2—O6	124.5 (2)	C13—C8—O2	118.7 (2)
O7—N2—C9	118.5 (2)	C9—C8—O2	121.1 (2)
O6—N2—C9	117.1 (2)	O4—N1—O3	123.7 (3)
C2—C3—C4	117.5 (2)	O4—N1—C2	119.0 (2)
C2—C3—H3	121.2	O3—N1—C2	117.2 (3)
C4—C3—H3	121.2	C5—C4—C3	120.8 (2)
C9—C10—C11	118.8 (2)	C5—C4—Cl1	120.7 (2)
C9—C10—H10	120.6	C3—C4—Cl1	118.6 (2)
C11—C10—H10	120.6	C13—C12—C11	121.0 (2)
C10—C9—C8	121.0 (2)	C13—C12—H12	119.5
C10—C9—N2	117.9 (2)	C11—C12—H12	119.5
C8—C9—N2	121.1 (2)	C1—C6—C5	120.5 (2)
C3—C2—C1	123.4 (2)	C1—C6—H6	119.8
C3—C2—N1	117.2 (2)	C5—C6—H6	119.8
C1—C2—N1	119.4 (2)	C4—C5—C6	120.6 (2)
C6—C1—C2	117.2 (2)	C4—C5—H5	119.7
C6—C1—C7	118.3 (2)	C6—C5—H5	119.7
C2—C1—C7	124.5 (2)	O1—C7—O2	123.6 (2)
C8—C13—C12	119.2 (2)	O1—C7—C1	125.7 (2)
C8—C13—H13	120.4	O2—C7—C1	110.53 (18)
C12—C13—H13	120.4	O5—C14—C11	125.2 (3)
C12—C11—C10	120.0 (2)	O5—C14—H141	121 (2)
C12—C11—C14	120.1 (2)	C11—C14—H141	114 (2)
C10—C11—C14	119.9 (2)		
			
C11—C10—C9—C8	0.3 (3)	C3—C2—N1—O4	170.3 (2)
C11—C10—C9—N2	179.76 (19)	C1—C2—N1—O4	−9.4 (4)
O7—N2—C9—C10	145.2 (2)	C3—C2—N1—O3	−13.2 (4)
O6—N2—C9—C10	−34.1 (3)	C1—C2—N1—O3	167.1 (3)
O7—N2—C9—C8	−35.4 (3)	C2—C3—C4—C5	−0.8 (4)
O6—N2—C9—C8	145.3 (2)	C2—C3—C4—Cl1	179.11 (18)
C4—C3—C2—C1	−0.3 (4)	C8—C13—C12—C11	0.9 (4)
C4—C3—C2—N1	−180.0 (2)	C10—C11—C12—C13	0.5 (4)
C3—C2—C1—C6	1.3 (4)	C14—C11—C12—C13	−179.9 (2)
N1—C2—C1—C6	−179.0 (2)	C2—C1—C6—C5	−1.4 (4)
C3—C2—C1—C7	−175.8 (2)	C7—C1—C6—C5	175.9 (2)
N1—C2—C1—C7	3.9 (4)	C3—C4—C5—C6	0.7 (4)
C9—C10—C11—C12	−1.1 (3)	Cl1—C4—C5—C6	−179.2 (2)
C9—C10—C11—C14	179.3 (2)	C1—C6—C5—C4	0.5 (4)
C12—C13—C8—C9	−1.6 (4)	C8—O2—C7—O1	−4.0 (3)
C12—C13—C8—O2	−176.0 (2)	C8—O2—C7—C1	179.86 (17)
C10—C9—C8—C13	1.0 (3)	C6—C1—C7—O1	−81.2 (3)
N2—C9—C8—C13	−178.4 (2)	C2—C1—C7—O1	95.9 (3)
C10—C9—C8—O2	175.29 (18)	C6—C1—C7—O2	94.8 (3)
N2—C9—C8—O2	−4.1 (3)	C2—C1—C7—O2	−88.1 (3)
C7—O2—C8—C13	−89.5 (3)	C12—C11—C14—O5	175.0 (3)
C7—O2—C8—C9	96.2 (2)	C10—C11—C14—O5	−5.4 (5)

### Hydrogen-bond geometry (Å, º)

**Table:**

*D*—H···*A*	*D*—H	H···*A*	*D*···*A*	*D*—H···*A*
C3—H3···O5^i^	0.93	2.44	3.289 (3)	151
C5—H5···O3^ii^	0.93	2.42	3.212 (4)	143
C12—H12···O6^iii^	0.93	2.58	3.317 (3)	137
C12—H12···O1^iv^	0.93	2.68	3.476 (3)	145

Symmetry codes: (i) *x*+1, *y*, *z*+1; (ii) *x*, *y*+1, *z*; (iii) *x*, *y*−1, *z*; (iv) −*x*+1, −*y*+1, −*z*.
