# Supplementary figures and images for: Crystal structure of 4-formyl-2-nitro­phenyl 4-chloro-2-nitro­benzoate
Source: Acta Crystallogr E Crystallogr Commun. 2015 Nov 14;71(Pt 12):o940. doi: 10.1107/S205698901502006X (PMC4719892; doi:10.1107/S205698901502006X)

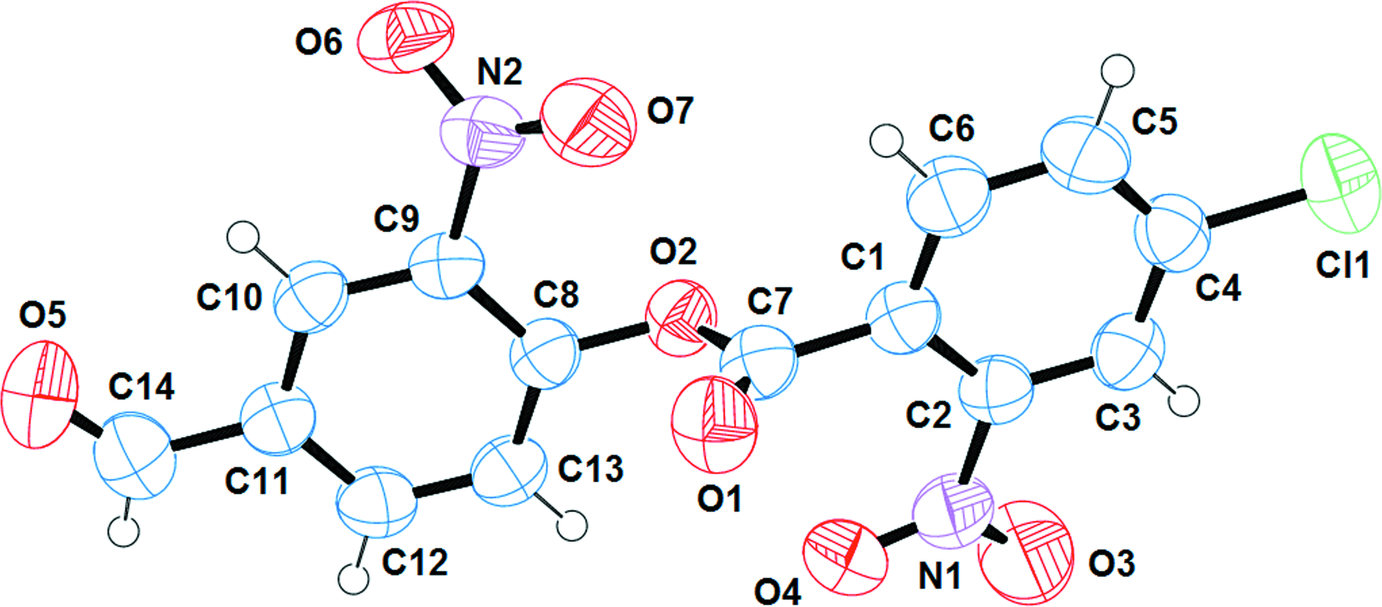

Supplement: Supplementary file 4 [file e-71-0o940-fig1.tif]

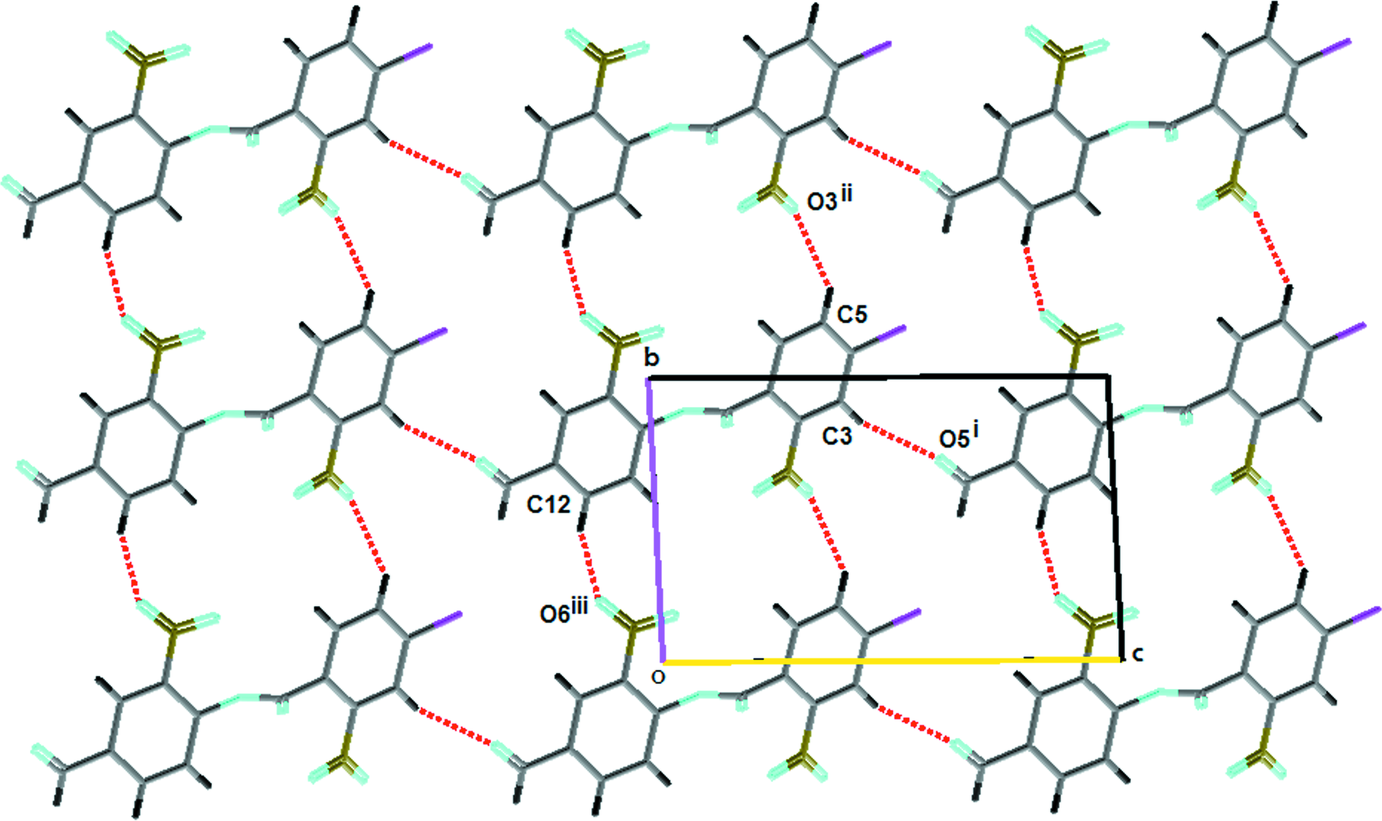

Supplement: Supplementary file 5 [file e-71-0o940-fig2.tif]

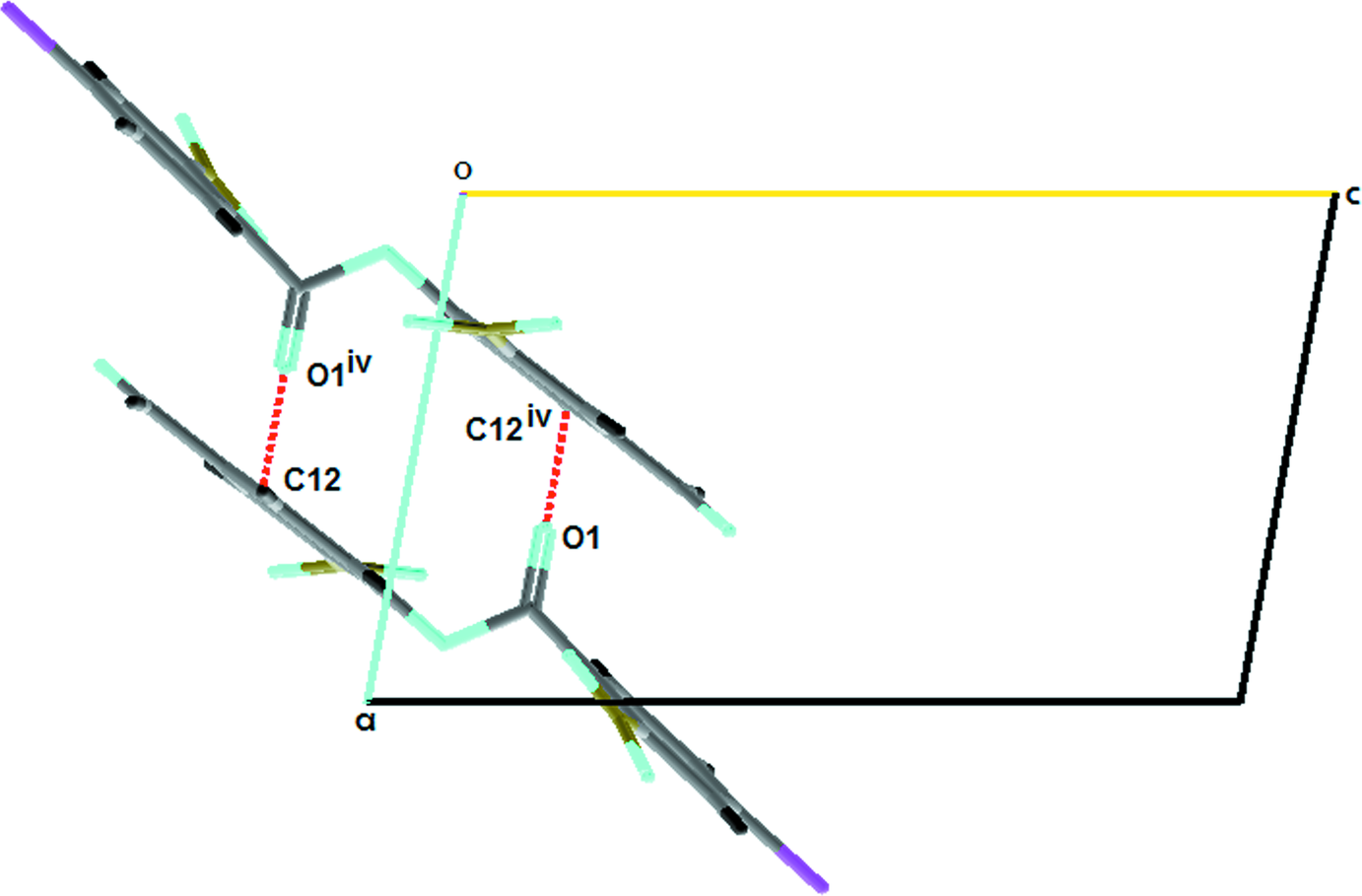

Supplement: Supplementary file 6 [file e-71-0o940-fig3.tif]
